# Determinants of decision-making among ever-married women in Indian households: A cross-sectional study based on binary logistic regression and multinomial logistic regression

**DOI:** 10.1371/journal.pone.0316805

**Published:** 2025-02-24

**Authors:** Riti Deshmukh, Sabina Bano, Sakshi

**Affiliations:** 1 Department of Geography, Banaras Hindu University, Varanasi, India; 2 Department of Geography, Mahila Maha Vidyalaya, Banaras Hindu University, Varanasi, India; Independent Consultant, UNITED STATES OF AMERICA

## Abstract

Empowerment of women is intrinsically linked to their participation in household decision-making, a crucial component for achieving gender equality and improving family well-being. Women’s decision-making is frequently cited as a proxy for empowerment and recognized as goal 5 of sustainable development goals. It remains a significant challenge in Indian households to achieve gender parity and poor concentration has been given in the studies of the Indian context. This study evaluates the types of decision-making among ever-married women in Indian households by investigating the socio-demographic factors that influence their role in household decisions. Utilizing data from the National Family Health Survey-5 (2019–21), which includes a sample of 51,758 women aged 15–49 years. This study employs a bivariate analysis to explore the association between predictive factors and women’s decision-making status. Before implementing a valid conclusion of multinomial logistic regression dealing with multinomial outcome variables, such as independent, joint, and dependent decisions, binary logistic regression was applied in the context of binary outcomes, specifically not making decisions alone and making decisions alone. Results reveal that only 3% of women make decisions independently. In contrast, 15% of women relied on dependently making decisions, and a majority of 82% of respondents reported jointly making decisions within their households. The conclusive model reveals that the likelihood of independent decision-making relative to joint decisions for rural women in India is 25% lower than for urban women, while dependent decision-making is 23% more often in rural areas than in urban ones as compared to jointly made decisions. Working women were 1.52 times more likely to make independent decisions, apart from that, the result indicates that 25% lower relative risk (RRR = 0.75, 95% CI = 0.69–0.81) of dependent decisions compared to joint ones. In contrast to the poorest households, women in the richest households are 42% less likely to make decisions independently as opposed to jointly. Regional variations are also evident, compared to women in northern regions, women from the South had the highest prevalence of independent decision-making power than joint decisions, with a relative risk ratio of 2.53 (RRR = 2.53, 95% CI= 2.04–3.14) and the lowest in central regions (RRR = 0.92, 95% CI= 0.73–1.17). Age emerges as a significant factor, compared to jointly making decisions, individuals in the age group of 35–45 have a relative risk ratio of 1.44 (RRR = 1.44, 95% CI= 1.18–1.77), and women over 45 years of 1.67 (RRR = 1.67, 95% CI=1.30–2.13) times greater autonomy than those in the age group below 25. Furthermore, compared to their counterparts who do not consume substances, women whose husbands do so have 1.44 (RRR = 1.44, 95% CI= 1.27–1.64) times higher probability of autonomy in making decisions relative to decisions made jointly. The study underscores the necessity for comprehensive educational programs, financial literacy workshops, improvement of transportation and healthcare decision-making, and region-specific cultural interventions among discriminatory castes by improving employment scenarios. Especially for rural women under the 25-age group can be a significant step in household decisions toward attaining gender equality.

## Introduction

Women’s empowerment is a critical determinant of social and economic development, and their participation in household decision-making processes serves as a fundamental indicator of this empowerment [1]. The term “empowerment” implies gaining control over one’s own power, while “women’s empowerment” refers to granting women the authority to make decisions across various areas of their lives [2]. Additionally, decision-making operates as a conduit of thinking, bridging the gap between rationally selected options and the eventual outcome for satisfying an individual life [3]. It is recognized as a cognitive behavioral indicator that positively correlates with power usage for operational activities in one’s livelihood, striking a balance between judgment and choices, and enhancing the efficacy of exploration capabilities towards the well-being condition of human life [4]. The state of personal well-being in any situation depends upon the sound of planned decision-making. In contrast, the challenges encountered by human society often arise from the repercussions of unplanned decisions in their activities [5]. In various spheres of human life, decision-making has recently been shaped by societal gender norms, and the equal contribution of women is perceived as a symbol of empowerment [2,6]. Because of societal norms pertaining to gender, women’s participation in decision-making varies significantly across social, cultural, economic, geographical, and political contexts [7]. However, women’s decision-making process is a pivotal indicator of their societal status, representing a crucial facet of power distribution with one’s own concerns in the social landscape [8]. According to subjective perceptions, when an individual exercises the capacity to make decisions without external influence, it is termed autonomy. Autonomy is commonly assessed through three dimensions; the ability to access and control resources, involvement in decision-making processes, and liberty of movement [9]. Specifically, the augmentation of their decision-making authority represents a fundamental stride in propelling women’s status forward through women’s empowerment and stands as a pivotal benchmark for measuring progress toward the objectives of sustainable development [8]. Spatially, in the context of any behavior in households, considered within the realm of private space, is sanctioned by either legal prescription or customary norms [10]. It reflects the idea that the actions and decisions made within the household are often shaped by the broader social, legal, and cultural frameworks that define acceptable conduct. Gender norms play a critical role in shaping the dynamics of decision-making within households, particularly in societies where traditional roles are deeply embedded. These norms dictate the division of labour, responsibilities, and authority between men and women, reinforcing a power hierarchy that limits women’s autonomy and decision-making capacity. According to Connell, gender norms are socially constructed expectations and behaviors associated with being male or female in a given society. These norms are perpetuated through socialization processes that begin in early childhood and are reinforced by family structures, educational systems, religious teachings, and media representations [11]. As a result, traditional gender norms often constrain women’s ability to make independent decisions within households, limiting their influence and reinforcing male dominance. Additionally, it was found in a study that India is a nation celebrated for its opulent cultural heritage and enduring traditions, where women are revered as deities [12]. Simultaneously, contemporary data reveals the pervasive abuse and unequal power structures affecting women, manifesting in mistreatment, violation, and disrespect. This degradation of women’s rights extends beyond public spheres and deeply permeates the confines of their homes, where instances of undermining women’s decision-making authority concerning external mobility, financial utilization, and health matters are rampant. Such intrusions not only strip women of their autonomy but also profoundly impact their mental and physical well-being, illustrating the pervasive nature of gender inequality in both private and public domains [13].

A recent pan-India survey namely National Family Health Survey-5 (NFHS-5) has reported that 71% of presently married women are engaged in decisions related to their healthcare, significant household expenditures, and visits to their family or relatives, either independently or jointly with their husbands, a slight increase from 63% as reported in National Family Health Survey-4 (NFHS-4). 11% of women do not participate in any of these three decision-making domains, compared to 16% in NFHS-4. Notably, women’s participation in decisions regarding major household purchases has risen from 73% in NFHS-4 to 80% in NFHS-5 [14]. Over four years since NFHS-4, a comparable increase of 6% in women’s participation in decisions about their healthcare (from 75% to 81%) and decisions concerning visits to family or relatives (from 75% to 81%). There has been an increase in women’s involvement in decision-making since NFHS-4.

The decision-making authority of women within households is linked to various aspects such as child health [8], family planning methods [12], reproductive health, parity levels [15], health issues [16], and the overall gender parity status of society [17]. Despite women constituting half of the population in India and being granted more rights than men, the condition of women remains distressing, particularly concerning the power dynamics in household decision-making [18]. An Indian study indicates that gender, serving as a benchmark for disparity, significantly influences dynamics within households. Furthermore, the research reveals a strong correlation between autonomy and the discriminatory status of women. For instance, the autonomy experienced by Indian women varies across regions, residences, social classes, castes, and other socio-demographic criteria [19]. Nevertheless, there is a specific requirement to analyze the prevailing status of decision-making collectively, autonomously, and interdependently, aiding in indirectly assigning the status of women’s empowerment [8,20].

Despite the availability of comprehensive cross-sectional data in India, research on women’s decision-making autonomy remains limited. Existing studies often use basic statistical methods, neglecting advanced techniques such as binary and multinomial logistic regression for analyzing factors linked to decision-making with dichotomous and multi-level outcomes [21,22**]**. They also overlook regional variations and the intersectionality of socio-demographic factors [1,23**]** and fail to thoroughly explore household dynamics, such as male partner influence and substance abuse [24**].** Although substantial research has explored women’s decision-making power from a health perspective and through the lens of autonomy, there is still a need for more detailed analysis. With this background, the primary objective is to discern the types of decisions taken by ever-married women with their respective background profiles in India and their association with women’s decision-making within their households.

Specifically, this study aims to address the gap by focusing on ever-married women aged 15–49 years, examining their decision-making regarding visits to family or relatives, major household purchases, utilization of their husband’s earnings, and their own healthcare. The study underscores the necessity for comprehensive educational programs, especially for rural women, and region-specific cultural interventions among discriminatory castes that raise awareness and empower women to exercise their decision-making power. Programs such as community-based education initiatives, financial literacy workshops, improved access to transportation and healthcare decision-making training could be tailored to these specific demographics. Enhancing women’s autonomy by improving employment scenarios can be a significant step in household decisions toward attaining gender equality and enabling women to contribute more effectively to India’s social and economic fabric. Additionally, policies promoting joint decision-making between spouses, particularly in healthcare and financial matters, may help reduce gender-based disparities in household decision-making authority.

## Materials and methods

### Data source

The study is based on the fifth round of data from the National Family Health Survey conducted in 2019–2021. This nationwide comprehensive survey (NFHS-5) interviewed 7,24,115 women and 1,01,839 men with response rates of 96.9% and 91.6%, respectively. The survey provides health and family well-being-related information, encompassing emerging issues in these domains such as fertility rates, infant and child mortality, maternal and child health, and various other health and family welfare indicators. This data is disaggregated by demographic characteristics at both national and state levels. The NFHS-5 surveys have been executed under the direction of the Ministry of Health and Family Welfare (MoHFW), Government of India. However, the NFHS-5 sample is designed as a stratified two-stage sample where Primary Sampling Units (PSUs) selection is based on the 2011 census, which serves as the sampling frame. In rural areas, PSUs were villages, and in urban areas, they were Census Enumeration Blocks (CEBs) were selected with probability proportional to size (PPS) systematic sampling methods. By combining household estimates, in each rural stratum, six substrata were formed by crossing three substrata and with two substrata, each created based on Schedule Caste/Schedule Tribe population percentages. Subsequently, PSUs were then organized by women’s literacy rates (age 6+). Apart from that, CEBs were sorted according to the percentage of the SC/ST population. Finally, 22 households were randomly chosen using systematic sampling in the last stage for each chosen rural and urban cluster. A comprehensive description of the survey process and sampling strategy is stated in the report of NFHS-5 [14].

### Ethics statement

The NFHS-5 survey (2019–21) protocol was reviewed and approved by the Institutional Review Boards of the International Institute of Population Sciences (IIPS) and ICF International. Each participant in the survey provided informed written consent prior to their involvement. The study is based on the Individual Recode (IR) data file, from NFHS-5 which is publicly available and complies with the Helsinki Declaration’s privacy and anonymity guidelines.

### Study design and sample size

In the current study, a cross-sectional data design was chosen, drawn from the National Family Health Survey (NFHS-5), the most recent Demographic and Health Survey in India. For our analysis, we evaluated individual recode (IR) file information from interviewed 7,24,115 ever-married women aged 15 to 49 from 28 states and 8 Union Territories providing information regards women’s health, socio-demographic background, empowerment status, and domestic violence. Of these women, only 76,611 responded to questions related to decision-making, which limited the sample size. To assess the degree of empowerment in decision-making, we focused on these 76,611 women, who were selected based on binary responses (Yes/No). Ultimately, to perform a multi-categorical analysis, a sample of 51,758 women was taken explicitly concerning decision-making and categorized into three groups: dependent, joint, and independent decision-makers.

### Dependent variable

The dependent variable of this study is the decision-making status of ever-married women aged 15 to 49. This variable is derived from the consideration of whether women engage in household decisions independently, jointly with their husbands, or dependently in four specific realms: (1) their own healthcare, (2) significant household purchases, (3) visits to their family or relatives and (4) decide what to do with money of husbands earn. These domains were selected from the women’s questionnaire of NFHS-5. Four selected questions on the decision-making of the questionnaire were taken into consideration. Who decides how your husband’s earnings will be used mainly you, mainly your husband, or you and your husband jointly or someone else? Who usually makes decisions about health care for yourself by means of mainly you, mainly your husband, you and your husband jointly, or someone else? Who usually makes decisions about making major household purchases like mainly you, mainly your husband, you and your husband jointly, or someone else? Who usually makes decisions about visits to your family or relatives for example mainly you, mainly your husband, you and your husband jointly, or someone else?. Firstly, responses were dichotomized into binary variables- “Not making the decision alone” has been coded as ‘0’ and “Makes alone decision” has been coded as ‘1’. Finally, to assess women’s empowerment status, responses were categorized into three distinct groups: women who made decisions independently of all of the four responses collectively were assigned a code of 1, those who made decisions jointly were assigned a code of 2, and those who indicated dependence in decision-making were assigned a code of 3, based on the information collected from the respondents.

### Independent variables

This study seeks to explore the relationship between decision-making and background factors among ever-married Indian women, utilizing a range of key socio-demographic and socio-economic variables. These variables were selected and incorporated based on previous research, with the goal of further analysing the logistic model [2,24,26–28]. These variables include ‘wealth index (Poorest, Poorer, Middle, Richer, Richest)’, ‘educational attainment (No education, Incomplete primary, Incomplete Secondary, Complete Secondary, Higher)’, ‘religion (Hindu, Muslim, Christian, Others)’, ‘respondent’s current working status (No, Yes)’, ‘educational level of the husband or partner (No education, Primary, Secondary, Higher, Don’t know)’, ‘caste group affiliation (Schedule caste, Schedule tribe, Other backward class (OBC), None of them, Don’t know’, ‘husband’s or partner’s alcohol consumption habits (No, Yes)’, ‘age group of the respondent (Below 25, 25–35, 35–45 and Over 45)’, ‘type of residence (Urban, Rural)’, and ‘geographical region (North, Central, East, Northeast, West, South)’.

### Methods of data analysis

This study employs descriptive statistics as a first step in the analysis to comprehend the background characteristics of ever-married women aged 15 to 49 in India. Subsequently, a chi-square analysis (χ2) is performed to examine the association between outcome and exposure variables. This preliminary check aids in excluding insignificant variables from the study, retaining only those with statistically significant results (p < 0.05). Additionally, an assessment of the Variance Inflation Factor (VIF) is conducted to detect multicollinearity in order to eliminate unstable and unreliable coefficient estimates from the regression model and to comprehend the substantial influence of independent variables on the meaningful conclusions as well as on predictor variables. Following that, binary logistics for binary replies (“Makes alone decision & Not making the decision alone”) and multinomial logistics for multiple response categories (Independently, Jointly, & Dependently) were used to provide interpretable coefficients characterizing the relationship between independent background factors and the non-sequential outcome variable. The results of the binary logistic model have been presented in the form of odds ratios (OR) and the multinomial logistic results are presented with the relative risk ratios (RRRs) with a 95% confidence interval (CI). Stata 16.0 statistical software was used to analyze the raw data [25].

## Results

### Profile of the respondents

Table 1 shows the background profile of the study samples. Among the 76,611 respondents, 82.51% of currently married Indian women do not make household decisions independently, while only 17.49% do. Notably, the highest proportion of women making decisions alone is observed among those aged over 45 years (20.84%), belonging to richer households (19.18%), having a high level of education (19.15%), being employed (23.13%), identifying as Christian (22.0%), residing in urban areas (20.25%), and living in the southern region (25.71%). Conversely, the highest proportion of women from the poorest households (84.87%), those aged under 25 years (87.67%), Muslims (85.29%), the unemployed (84.47%), rural residents (83.75%), and those from central India (88.35%) have found not making decision independently.

**Table 1:** Profile of respondents and prevalence of decision making across background characteristics.

Background variables	Total number of respondents		Making decisions alone (Binary responses) (f)	
	(n)	%	No (%)	Yes (%)
**Wealth Index**				
Poorest	14,317	18.68	84.87	15.13
Poorer	15,570	20.32	84.14	15.86
Middle	15,734	20.53	81.67	18.33
Richer	15,652	20.43	80.82	19.18
Richest	15,338	20.02	81.26	18.74
**Current Age (In years)**				
Below 25	16,272	21.24	87.67	12.33
25–35	29,389	38.36	82.87	17.33
35–45	23,161	30.23	79.56	20.44
Over 45	7,789	10.16	79.16	20.84
**Educational Attainment**				
No education	20,992	27.40	83.05	16.95
Incomplete Primary	10,608	13.85	82.07	17.93
Incomplete Secondary	33,882	44.23	82.74	17.26
Complete secondary	1,182	1.54	84.31	15.69
Higher	9,947	12.98	80.85	19.15
**Religion**				
Hindu	62,158	81.13	82.11	17.89
Muslim	10,588	13.82	85.29	14.71
Christian	1,656	2.16	78.00	22.00
Others	2,209	2.88	83.94	16.06
**Caste**				
Schedule caste	16,357	22.49	81.94	18.06
Schedule tribe	6,977	09.59	83.62	16.38
Other backward classes (OBC)	33,554	46.14	82.27	17.73
None of them	15,294	21.03	82.39	17.61
Don’t know	545	0.75	85.92	14.08
**Working status**				
No	56,891	74.26	84.47	15.53
Yes	19,720	25.74	76.87	23.13
**Husbands’ or Partner’s education level**				
No education	12,633	16.49	81.94	18.06
Primary	11,173	14.58	81.97	18.03
Secondary	40,385	52.71	82.58	17.42
Higher	12,188	15.91	83.32	16.68
Don’t know	232	0.30	85.96	14.04
**Husbands or Partners drink alcohol**				
No	45,452	76.01	83.88	16.12
Yes	14,344	23.99	76.94	23.06
**Type of Residence**				
Urban	23,760	31.01	79.75	20.25
Rural	52,851	68.98	83.75	16.25
**Geographical Region**				
North	10,804	14.10	83.93	16.07
Central	17,618	22.99	88.35	11.65
East	18,579	24.25	85.32	14.68
Northeast	2,872	03.75	86.90	13.10
West	10,816	14.12	77.70	22.30
South	15,922	20.78	74.29	25.71

n, sample of ever-married women for each background; f, frequency of ever-married women making decisions in the household; All % are weighted.

In general, women from the poorest households constitute approximately 19% of the female population. The majority of Indian women are aged 25–35 and 35–45, accounting for around 38.36% and 30.23%, respectively. Women aged over 45 years comprise approximately 10% of the female population.

In India, around 27% of women lack any formal education, whereas highly educated women constitute about 13%. Most of the women from the Hindu religion account for around 81.13%. A large proportion of women, around 46%, comes from the Other Backward Classes. Approximately 74.26% of women are not formally working. The result reveals that nearly one-fourth of women’s spouses are alcohol consumers.

About 53% of women’s spouses have completed only secondary education. Women from rural areas make up around 69% of the population, compared to 31% in urban areas. Around one-fourth of women are from the eastern zone, comprising about 24.25% of the total female population followed by, women from Central (22.99%), South (20.78), West (14.12%), North (14.10%), and Northeast (03.75%) regions.

### Prevalence of decision-making among women in India with socio-demographic characteristics

Table 2 presents the distribution of decision-making status among married women in India across their background characteristics. Along with that, a comprehensive bi-bivariate analysis was conducted among 51,758 ever-married women in India from an initial pool of 76,611 women evaluated for sample selection in the decision-making study. The selected group was assessed based on their independent, joint, and dependent decision-making responsibilities in areas such as healthcare, mobility, household purchases, and preferences regarding their husband’s income from the variables of Making decisions alone with binary responses (Yes/No).

**Table 2:** Results from the bi-variate analysis represent the association between determinants with decision-making among ever-married women.

Background variables	n	n(%)	Prevalence of decision-making among ever-married women (f)			Chi-square	P value
			Independent decision (1)	Jointly decision (2)	Dependent decision (3)		
**Wealth Index**						83.73	<0.001
Poorest	10,204	19.72	03.72	80.71	15.57		
Poorer	10,751	20.77	03.30	79.85	16.86		
Middle	10,492	20.27	04.07	80.36	15.56		
Richer	10,150	19.61	03.47	81.86	14.67		
Richest	10,161	19.63	02.31	86.06	11.63		
**Current age (In Years)**						609.04	<0.001
Below 25	11,018	21.29	01.87	76.08	22.05		
25–35	19,955	38.55	03.04	82.33	14.63		
35–45	15,538	30.02	04.39	84.45	11.16		
Over 45	5,247	10.14	04.82	83.34	11.84		
**Educational attainment**						160.54	<0.001
No education	14,548	28.11	04.09	79.58	16.33		
Incomplete primary	7,271	14.05	03.69	81.91	14.39		
Incomplete Secondary	22,515	43.50	03.00	81.63	15.37		
Complete Secondary	773	1.49	02.25	81.00	16.75		
Higher	6,651	12.85	02.88	86.74	10.39		
**Religion**						195.95	<0.001
Hindu	41,922	81.00	3.51	82.03	14.46		
Muslim	7,163	13.84	2.74	78.56	18.71		
Christian	1,099	2.12	4.21	84.88	10.90		
Others	1,574	3.04	2.22	86.25	11.53		
**Caste**						97.31	<0.001
Schedule caste	11,119	22.64	3.93	81.02	15.05		
Schedule tribe	4,812	9.80	2.59	83.56	13.85		
OBC	22,509	45.83	3.43	81.12	15.45		
None of them	10,284	20.94	3.17	83.60	13.23		
Don’t know	386	0.79	3.31	75.24	21.45		
**Working status**						293.92	<0.001
No	38,898	75.15	2.69	81.21	16.10		
Yes	12,860	24.85	5.47	83.34	11.20		
**Husband or partner’s education level**						185.57	<0.001
No education	8,748	16.90	5.27	78.15	16.58		
Primary	7,620	14.72	3.81	80.95	15.23		
Secondary	27,055	52.27	3.07	82.02	14.91		
Higher	8,175	15.80	1.93	85.52	12.55		
Don’t know	160	0.31	5.34	74.05	20.61		
**Husband or partner drinks alcohol**						87.13	<0.001
No	31,257	77.27	2.69	83.51	13.79		
Yes	9,196	22.73	5.43	80.22	14.35		
**Type of Residence**						73.64	<0.001
Urban	15,783	30.49	3.81	84.04	12.15		
Rural	35,976	69.51	3.19	80.73	16.08		
**Region**						510.12	<0.001
North	7,229	13.97	1.93	82.67	15.39		
Central	13,009	25.13	2.26	82.64	15.09		
East	13,219	25.54	3.26	82.57	14.17		
Northeast	2,102	4.06	2.20	87.89	9.91		
West	6,753	13.05	3.57	83.48	12.95		
South	9,446	18.25	6.31	76.00	17.69		

n, sample of ever-married women for each background; f, frequency of ever-married women making decisions in the household; All % are weighted.

A majority of 82% of respondents reported jointly made decisions within their households. In contrast, 15% of women reported making decisions dependently, while only 3% reported making decisions independently.

The analysis from the chi-square test reveals that women’s decision-making status is substantially correlated with their age group, level of education, wealth, religion, working status, caste, husband or partner’s educational achievement, Husband or partner alcohol intake practice, residence type and geographical location with statistical significance(p=<0.001).

The results illustrate that age has a positive relationship with independent decision-making. Notably, adult women over the age of 45 have a higher level of active engagement in independent decision-making, accounting for 4.82%. In comparison, the lowest is approximately 2% among those under 25. On the other hand, dependent decision-making stands at 11. 84% in the age bracket over 45, contrasting patterns identified in the age group below 25 (22.05%). Concerning education, Women without formal education prefer independent decision-making, accounting for 4.09%. Conversely, 86.74% of highly educated women prefer joint household decision-making processes. The largest percentage of independent decision-making is reported among Christian women (4.21%), followed by Hindu women (3.51%), Muslims (2.74%), and others (2.22%). In terms of dependent decision-making, the highest percentage recorded by Muslim women was 18.71%, followed by 14.46% by Hindu women, and the lowest recorded by Christian women (10.90%). The wealth index shows a negative trend in which women from the most economically disadvantaged families are inclined to make decisions independently (around 4%), whereas those from the wealthiest families have only 2%. In the context of joint decision-making, the tendency reverses, with the richest women making the majority of decisions collaboratively (86.06%). In terms of social class category, scheduled caste women were found more actively involved in independent decision-making than women from other cases where Scheduled caste women account for approximately 4% of independent decision-making, whereas scheduled tribe women were found lowest percentage at 2.59%. The findings demonstrate a relationship between the working status of women and exercising autonomy in decision-making. Independent decisions taken by working women account for 5.47%, while the jointly taken decision percentage is 83.34% and the dependently taken percentage is 11.20%. On the contrary, non-working women prefer joint and dependent decision-making, with a significant rate of 81.21% and 16.10%, respectively. Considering as the husbands’ educational attainment rises, the percentage of independent decision-making among women decreases noticeably. In households with highly educated husbands, just 1.93% of women actively participate in independent decision-making, compared to 5.27% in households where husbands are formally uneducated. In contrast, when the husband is well-educated, the incidence of joint decision-making is higher (85.52%) than when the husband is uneducated (78.15%). Women whose spouses are prone to alcohol consumption are more likely to make independent decisions, accounting for 5.43%, as compared to non-alcoholic partners (2.69%). In terms of geographic distribution, women in the southern region display a preference for independent decision-making at a rate of 6.31%, surpassing the rest of the country while 76% of the women tend to make decisions jointly with their partner. Women from the northeast region were valued over the dependent and joint decisions at a percentage of 9% and 87%, respectively.

Urban women largely depended on joint decisions, representing 84.04% while their rural counterparts’ joint and dependent decision-making is major, accounting for 80.73 and 16.08%.

### Estimates from the binary logistic regression analysis

Separate logistic regression models incorporating all the statistically significant variables were fitted with Decision-making as the outcome variable. Table 3 depicts the unadjusted and adjusted odds ratios obtained from the binary logistic regression model. An unadjusted odd ratio reveals individual correlation of the predictor variables with decision making, while adjusted reveals adjustment effects of exposure on the outcome by controlling other variables. An OR greater than 1 indicates an increased chance of the outcome, while an OR less than 1 indicates a decreased chance. An OR of 1 means there’s no association between outcome and exposures. Binary logistic analysis reveals that factors such as a woman’s age, education level, wealth status, employment status, and her husband’s alcohol consumption significantly influence her likelihood of participating in household decision-making independently. The adjusted analysis did not portray any significant relationship between women’s decision-making status and caste or the educational attainment of their husbands or partners. Women’s likelihood to make independent decisions increases with advancing age. Notably, women over the age of 45 years showed a 75% higher tendency for autonomous decision-making than their younger counterparts. Rural women are 19% less likely to make decisions than urban women alone. The result of the study showed a significant negative relationship between women’s alone decisions and their level of wealth. Women from the richest households made 16% less decisions than the poorest.

**Table 3:** Binary regression model for decision-making among ever-married Indian women.

Background variables	Unadjusted odd ratio (UOR)		Adjusted odd ratio (AOR)	
	OR (95% CI)	P value	OR (95% CI)	P value
**Wealth Index**				
Poorest	Ref.		Ref.	
Poorer	1.07 (1.00–1.14)	0.039	0.94 (0.87–1.02)	0.134
Middle	1.28 (1.20–1.36)	<0.001***	0.96 (0.88–1.04)	0.344
Richer	1.34 (1.26–1.43)	<0.001***	0.89 (0.81–0.98)	0.015**
Richest	1.30 (1.22–1.39)	<0.001***	0.84 (0.75–0.93)	0.001***
**Current age (In Years)**				
Below 25	Ref.		Ref.	
25–35	1.46 (1.38–1.55)	<0.001***	1.33 (1.24–1.43)	<0.001***
35–45	1.84 (1.74–1.96)	<0.001***	1.66 (1.54–1.79)	<0.001***
Over 45	1.94 (1.80–2.09)	<0.001***	1.75 (1.58–1.93)	<0.001***
**Educational attainment**				
No education	Ref.		Ref.	
Incomplete primary	1.09 (1.02–1.16)	0.011	1.08 (1.00–1.17)	0.042
Incomplete Secondary	1.02 (0.97–1.07)	0.436	1.12 (1.04–1.19)	0.002
Complete secondary	0.93 (0.79–1.10)	0.412	0.94 (0.77–1.15)	0.561
Higher	1.20 (1.12–1.28)	<0.001***	1.34 (1.21–1.49)	<0.001***
**Religion**				
Hindu	Ref.		Ref.	
Muslim	0.79 (0.74–0.84)	<0.001	0.91 (0.84–0.99)	0.026**
Christian	1.37 (1.22–1.55)	<0.001	1.00 (0.87–1.16)	0.922
Others	0.90 (0.80–1.02)	0.092	0.84 (0.73–0.97)	0.020**
**Caste**				
Schedule caste	Ref.		Ref.	
Schedule tribe	0.87 (0.80–0.94)	<0.001**	0.87 (0.80–0.96)	0.005
OBC	0.97 (0.92–1.02)	0.239	0.94 (0.88–1.00)	0.061
None of them	0.96 (0.91–1.02)	0.205	1.01 (0.94–1.09)	0.712
Don’t know	0.76 (0.59–0.98)	0.033	0.89 (0.67–1.19)	0.468
**Working status**				
No	Ref.		Ref.	
Yes	1.61 (1.55–1.68)	<0.001***	1.28 (1.22–1.35)	<0.001***
**Husband or partner’s education level**				
No education	Ref.		Ref.	
Primary	0.98 (0.91–1.05)	0.536	0.98 (0.90–1.06)	0.658
Secondary	0.95 (0.90–1.00)	0.054	0.95 (0.88–1.03)	0.214
Higher	0.92 (0.85–0.98)	0.017	0.85 (0.77–0.95)	0.003**
Don’t know	0.77 (0.52–1.13)	0.182	0.81 (0.51–1.29)	0.378
**Husband or partner drinks alcohol**				
No	Ref.		Ref.	
Yes	1.59 (1.51–1.67)	<0.001***	1.51(1.43–1.59)	<0.001***
**Type of Residence**				
Urban	Ref.		Ref.	
Rural	0.77 (0.73–0.80)	<0.001***	0.81 (0.77–0.86)	<0.001***
**Region**				
North	Ref.		Ref.	
Central	0.66 (0.61–0.71)	<0001***	0.65 (0.59–0.71)	0.002***
East	0.88 (0.82–0.94)	<0.001***	0.87 (0.79–0.95)	<0.001***
Northeast	0.81 (0.72–0.92)	0.001***	0.75 (0.63–0.88)	<0.001***
West	1.48 (1.37–1.58)	<0.001***	1.40 (1.29–1.53)	<0.001***
South	1.81 (1.69–1.92)	<0.001***	1.59 (1.46–1.72)	<0.001***

Ref., reference category; CI, confidence interval; Sig., significance *** p < .01, ** p < .05, * p < .1

Education emerges as a pivotal indicator, revealing that highly educated women are 1.34 times more likely to make decisions alone in the realms of healthcare, mobility, household expenditures, and the utilization of funds earned by their husbands. The odds of women’s autonomy are 9% lower for Muslim women and 16% lower for women from other religions when contrasted with their Hindu counterparts. Working women reported being 1.28 times more involved in making decisions alone than the non-working women. The geographical region stands out as a significant factor, with a 40% and 59% higher likelihood of making decisions autonomously in the western and southern regions of India, respectively, as compared with northern India. A negative correlation has been found between women’s independence in decision-making and husbands’ education. For instances where husbands are alcoholic, women are 1.51 times more likely to be autonomous compared to cases where husbands do not intake alcohol.

### Correlates of decision making

The conclusive model of multinomial logistic regression, showing the odds associated with decision-making across various exposure variables has been listed in (Table 4). In general, if the RRR is more than one, the outcome is more likely to be in the group of comparison, and if the RRR is less than one, it is less likely to be in the reference group. The substantial association between women’s involvement in decision-making and socio-economic background characteristics is highly significant. The model illustrates a significant association between women’s type of decision-making and various background factors, including current age, wealth Index, educational attainment, religion, husband or partner’s educational level, husband or partner’s alcoholic status, type of residence, and geographical region.

**Table 4:** Multinomial Logistic Regression, the correlation between independent, joint, and dependent decision-making with their background characteristics.

Background variables	Independent decision		Dependent decision	
	RRR (95% CI)	P value	RRR (95% CI)	P value
**Wealth Index**				
Poorest	Ref.		Ref.	
Poorer	0.90 (0.76–1.08)	0.207	1.06 (0.97–1.17)	0.160
Middle	0.89 (0.73–1.08)	0.231	0.93 (0.84–1.03)	0.187
Richer	0.79 (0.63–1.00)	0.053**	0.99 (0.88–1.10)	0.816
Richest	0.58 (0.44–0.78)	<0.001***	0.92 (0.80–1.05)	0.212
**Current age (In Years)**				
Below 25	Ref.		Ref.	
25–35	1.10 (0.91–1.34)	0.533	0.66 (0.61–0.71)	<0.001***
35–45	1.44 (1.18–1.77)	<0.001***	0.50 (0.46–0.55)	<0.001***
Over 45	1.67 (1.30–2.13)	<0.001***	0.47 (0.41–0.54)	<0.001***
**Educational attainment**				
No education	Ref.		Ref.	
Incomplete primary	0.98 (0.81–1.18)	0.862	0.72 (0.66–0.80)	<0.001***
Incomplete Secondary	0.95 (0.79–1.12)	0.604	0.69 (0.64–0.76)	<0.001***
Complete secondary	0.76 (0.45–1.43)	0.390	0.76 (0.59–0.97)	0.030**
Higher	1.09 (0.79–1.41)	0.520	0.51(0.43–0.58)	<0.001***
**Religion**				
Hindu	Ref.		Ref.	
Muslim	1.05 (0.84–1.30)	0.646	1.24 (1.13–1.37)	<0.001***
Christian	1.12 (0.82–1.35)	0.359	0.69 (0.59–0.82)	<0.001***
Others	1.52 (1.13–1.87)	0.001***	1.04 (0.90–1.20)	0.589
**Caste**				
Schedule caste	Ref.		Ref.	
Schedule tribe	0.76 (0.62–0.93)	0.009**	0.97 (0.89–1.00)	0.623
OBC	0.89 (0.75–1.04)	0.140	0.98 (0.96–1.06)	0.691
None of them	0.96 (0.78–1.18)	0.756	0.94 (0.97–1.10)	0.244
Don’t know	1.09 (0.52–2.26)	0.810	1.60 (1.17–2.22)	0.004***
**Working status**				
No	Ref.		Ref.	
Yes	1.52 (1.34–1.73)	<0.001***	0.75 (0.69–0.81)	<0.001***
**Husband or partner’s education level**				
No education	Ref.		Ref.	
Primary	0.71 (0.58–0.86)	<0.001***	0.91 (0.81–1.00)	0.073
Secondary	0.64 (0.54–0.76)	<0.001***	0.91 (0.82–0.99)	0.037**
Higher	0.51 (0.39–0.68)	<0.001***	1.00 (0.87–1.14)	0.988
Don’t know	0.71 (0.25–1.94)	0.503	0.72 (0.41–1.28)	0.275
**Husband or partner drinks alcohol**				
No	Ref.		Ref.	
Yes	1.44 (1.27–1.64)	<0.001***	0.96 (0.89–1.03)	0.248
**Type of Residence**				
Urban	Ref.		Ref.	
Rural	0.75 (0.65–0.88)	<0.001***	1.23 (1.14–1.34)	<0.001***
**Region**				
North	Ref.		Ref.	
Central	0.92 (0.73–1.17)	0.515	0.86 (0.79–0.94)	0.002***
East	1.10 (0.87–1.39)	0.409	0.70 (0.63–0.78)	<0.001***
Northeast	1.44 (1.12–1.86)	0.004**	0.72 (0.63–0.82)	<0.001***
West	1.75 (1.37–2.24)	<0.001***	0.97 (0.86–1.09)	0.564
South	2.53 (2.04–3.14)	<0.001***	1.49 (1.35–1.66)	<0.001***

RRR, relative risk ratio; CI, confidence interval.

***p < 0.01.

**p < 0.05.

*p < 0.10.

In this model, it was determined that independent decision-making inversely correlated with the wealth index of women. With statistical significance, women from the wealthiest households showed a 0.42 times lower relative risk ratio (RRR = 0.58, 95% CI = 0.44–0.78) of making independent decisions in comparison to those making joint decisions. Surprisingly, the results indicate that age is a pivotal determinant of decision-making. The probability of independent decisions was 1.44 and 1.67 times more among individuals aged 35–45 and those above 45 with a relative risk ratio of (RRR = 1.44, 95% CI = 1.18–1.77; RRR = 1.67, 95% CI = 1.30–2.13) respectively, compared to those who make joint decisions. On the other, the likelihood of making dependent decisions was 44%, 50%, and 53% lowering the relative risk ratio (RRR = 0.66, 95% CI = 0.61–0.71; RRR = 0.50, 95% CI = 0.46–0.55; RRR = 47, 95% CI = 0.41–0.54) among individuals aged 25–35, 35–45, and above 45, respectively compared with the jointly taken decision. The probability of having a dependent decision were 0.72, 0.69, and 0.51 times reduced relative risk ratio (RRR = 0.72, 95% CI = 0.66–0.80; RRR = 0.69, 95% CI = 0.64–0.76; RRR = 0.51, 95% CI = 0.43–0.58) respectively among those women who have done incomplete primary education, incomplete secondary education, and higher education compared with the jointly taken decision. It is interesting to note that compared with uneducated women educated women are significantly less likely to participate in dependent decision-making (p < 0.001) about their health care, household purchases, visits to family and relatives, and what to do with money their husband earns than jointly decision after adjustment for other factors. In addition, after controlling other exposures, no significant relationship has been found between educational attainment and independent decision-making as opposed to joint decision-making. Excluding individuals who identify with Hinduism, Islam, and Christianity, other religious women had a 1.52 times higher relative risk ratio of making independent decisions (RRR = 1.52, 95% CI = 1.13–1.87) than those who made joint decisions. Conversely, the relative risk ratio of having dependent decisions was 1.24 times higher and 0.31 times lower relative risk ratio for women adhering to the Muslim and Christian faiths, respectively, in comparison to those who made joint decisions. Caste plays a crucial role in influencing the prevalence of decision-making status. In contrast to women from the scheduled caste, those from the scheduled tribe exhibited a lower relative risk ratio of engaging in independent decision-making (RRR = 0.76, 95% CI = 0.62–0.93) with statistical significance compared with joint decisions. The correlation between working status and autonomy in decision-making is noteworthy. Women who are employed demonstrated a 1.52 times higher relative risk ratio of making independent decisions and with statistical significance, among working women, revealed a 25% reduction of the relative risk ratio (RRR = 0.75, 95% CI = 0.69–0.81) of dependent decisions compared to joint decisions. Furthermore, the educational level of the husband or partner plays considerable significance in the sphere of women’s decision-making within the household. The educational status of husbands is inversely correlated with autonomy in the decision-making context for women. Women with highly educated husbands or partners reduced the 49% relative risk ratio (RRR = 0.51, 95% CI = 0.39–0.68) of making independent decisions compared with joint decisions. But in the context of dependent decision making, women with highly educated partners have shown (RRR = 1.00, 95% CI = 0.87–1.14) insignificant results (p = 0.988). With the statistical significance, the model indicates that women with alcoholic partners or husbands were more prone to raise the relative risk ratio (RRR = 1.44, 95% CI = 1.27–1.64) of engaging in independent decision-making as opposed to joint decisions and reduce the relative risk ratio of engaging dependent decision (RRR = 0.96, 95% CI = 0.89–1.03) with statistical non-significant. It is evident that the residential setting also plays a vital role in the context of women’s decision-making. The probability of independent decision-making is 25% reducing the relative risk ratio (RRR = 0.75, 95% CI = 0.65–0.88) in rural areas than urban counterparts compared with decisions made jointly. Conversely, within the sphere of dependent decision-making among rural women, the relative risk ratio is increased by 23% (RRR = 1.23, 95% CI = 1.14–1.34) compared with the joint decision. Results reveal that geographical variation has a significant impact on the status of decision-making among ever-married Indian women. Women from Northeast, West, and South India were more likely to engage in independent decision-making with a relative risk ratio of (RRR = 1.44, 95% CI = 1.12–1.86; RRR = 1.75, 95% CI = 1.37–2.24; RRR = 2.53, 95% CI = 2.04–3.14). Conversely, women from Central, East, Northeast, and West regions were less likely to make dependent decision-making as opposed to joint decision-making with relative risk ratios of (RRR = 0.86, 95% CI = 0.79–0.94; RRR = 0.70, 95% CI = 0.63–0.78; RRR = 0.72, 95% CI = 0.63–0.82; RRR = 0.97, 95% CI = 0.86–1.09) respectively.

## Discussion

The study examined the decision-making status as a key measure of women’s empowerment and aimed to identify its potential determinants among ever-married women aged 15–49 in Indian households. The study’s findings reveal that 3.38% of women independently make decisions, 81.74% engage in joint decision-making, and 14.88% rely on dependent decision-making. Subsequently, binary logistic and multinomial logistic regression analyses were used to measure the association between the outcomes and exposure variables. The model reveals that age, employment status, and having an alcoholic partner are significant predictors of autonomy in household decision-making. Conversely, advancing age, higher educational attainment, religious affiliation, employment, rural residency, and regional disparities are strongly correlated with participation in dependent decision-making.

In the decision-making landscape of ever-married women, age emerges as a pivotal factor, specifically in the context of healthcare facilities [17]. As women age, their cumulative experiences with childbirth and marital roles become more ingrained, influencing their decision-making processes. In varying socio-economic contexts, older women often demonstrate a stronger sense of loyalty and adaptability in navigating post-marital life, contrasting with the behaviours typically observed among younger women. This trend is not different in India but is also evident in other developing countries like Nepal, Ethiopia, and other South Asian nations [26,29]. The accumulation of life experiences with advancing age enhances women’s confidence in their abilities, leading to greater assertiveness in expressing their opinions which becomes respective from all directions [30].

Using multi-nominal logistics, the current study illustrates that educational achievement does not invariably serve as an unequivocal advocate for fostering participation in independent decision-making. Instead, the findings unveil a negative correlation between educational attainment and involvement in dependent decision-making. However, in the binary logistic model, a pattern similar to that observed in Nigeria and Ethiopia emerges, where higher education consistently increases awareness of one’s rights and life responsibilities in the context of decision-making [29,31,32].

Additionally, better employment and financial stability are made possible by education, which gives women more options in life and helps to create confidence of making decisions in households [31]. The reasons behind the disparity in educational achievement remain unclear and require further research for a better understanding.

This research affirms that women in employment demonstrate an inclination to engage in household decision-making independently [17,29]. Employee women because of their economic involvement and their participation in social interactions outside the home are more significant contributor for their decisions and values in households [33]. Furthermore, it is observed that working women participate in dependent decision-making in only less than 25% of cases [32]. This may be attributed to their economic dependence on partners, as a lack of financial independence undermines women’s confidence in their own existence regarding the decision context of households [29].

The results of the study indicated a significant correlation between wealth status and women’s decision-making abilities. The finding reveals that as women become wealthier, they are less likely to participate in independent decisions, substantiated by earlier research done in Nepal [26]. However, in India, the fundamental cause may be that ground-rooted patriarchy for property ownership limits women’s participation in economic settings and their ability to make decisions [33].

The result of the study suggests that the educational level of husbands emerges as an essential factor in household decision-making. In the realm of spousal or partner education, the autonomy of women in decision-making becomes less consequential when husbands possess advanced educational credentials, in contrast to those with no formal education. Similarly, a study conducted in China found that highly educated husbands wield more power, indicating a greater gender disparity in household decision-making [34]. Therefore, further research is required to understand the complex relationship between the education of partners or husbands and women’s autonomy in decision-making.

Rural women are significantly less likely to take part in independent decision-making than urban women. Urban women exhibit greater awareness of their rights and societal status due to extensive exposure to mass media, rendering them more cognizant of the advantages in their contemporary environment compared to rural women. This observation is consistent with findings from a study conducted in Ethiopia, in the context of health which substantiate the study’s results [29]. However, our study finding confirms that in the context of dependent decision-making rural women are more active rather than urban women. It is apparent that monetary contribution and its association with decision-making are straightforward, but in general, rural women are less exposed and assessed to participate in paid work in the monetary context, which increases the likelihood of decision-making dependency [35].

In contrast to other regions, women in South India exhibit a higher degree of autonomy in household decision-making. Various indicators, such as advancement in women’s education, the scope of their involvement in different agencies, and marital behaviour in southern India, can be attributed as contributing factors to the prevalence of women’s autonomy in this region [9,36]. In the western regions, specifically, Maharashtra and Gujrat, the enhancement of women’s rising power is shaped by elements including organizational endeavors, the engagement of local governance, and the dynamics of migration [37,38]. Additionally, the study ensures that regarding socio-economic context women in the northeastern region of India generally enjoy a higher status compared to other parts of the country and are 1.44 times more likely to exercise autonomy in decision-making. However, entrenched cultural and maternal customary traditions continue to perpetuate significant reduction in gender-based disparities in northeastern societies, rather than discrimination, particularly for patriarchal systems [39].

The study highlights that husbands’ or partners’ alcoholic tendencies result in increased physical and mental dependence on their wives, leading to instability in their overall lifestyle, especially when it comes to making life decisions. In this context, wives tend to assume greater autonomy in making decisions about their lives within the household. Furthermore, in the Indian context, marital discord, economic insecurity, and social discrimination stand out as prevalent factors contributing to alcohol addiction among spouse’s increased dependency on their life decisions on their partners [40].

The study grapples with a few limitations. While decision-making is a widely employed concept, our study encompasses only four variables—health, household expenditure, utilization of husbands’ money, and women’s mobility—extracted from the NFHS-5 dataset which restricts our ability to identify causal relationships between predictor and outcome variables. This limited scope necessitates further exploration to comprehensively capture the intricacies of decision-making. Additionally, our study’s confinement to women in the 15–49 age group represents another drawback, as it fails to encompass the entirety of the female population. More in-depth surveys and research are necessary to clarify how regional and cultural differences in India impact women’s decision-making. Additionally, the influence of women’s education and husbands’ education on decision-making remains unclear, especially in the context of urban and rural settings. In addition to education, economic empowerment is vital. Skill development programs, employment opportunities, and financial incentives for women entrepreneurs can provide the economic independence needed to assert decision-making authority within households. Addressing regional disparities is equally crucial. Tailored interventions that respect cultural contexts, combined with partnerships with local organizations, can encourage independent decision-making among women in underrepresented areas, particularly in rural and economically disadvantaged regions.

## Conclusion

The study demonstrated that as a proxy for empowerment, decision-making in the context of households is an essential measure of gender inequality implied by power distribution. Findings indicate that only 3.38% of Indian women participate in decision-making independently, with a significant percentage of 81.74% participating in joint decision-making and 14.88% dependent on others for making decisions. Additionally results reveals that women’s age, educational level, employment status, husbands’ or partners’ education, alcohol intake habit by partners or husbands, residential setting, and geographic location all variables significantly influence on the types of women’s decision-making. The study uncovers that woman over the age of 45, working, from southern regions, and husbands’ alcohol consumption all have a significant impact on women’s autonomy in making household decisions. Notably, rural women from wealthier households with highly educated husbands are less likely to make decisions independently, highlighting the complex interplay between socio-economic factors and gender dynamics. Aside from that, dependent decision-making has been found integrally linked to women’s age, education, and rural residential settings. The study was unable to determine the reasons behind geographical variations in decision-making or the role of education in these processes properly, highlighting the need for a comprehensive and in-depth analysis to uncover these underlying relationships. However, the study emphasizes the importance of laws and policies that promote gender equality by empowering women in household decision-making roles. The findings of this study underscore the critical need for targeted policy interventions to promote gender equality and empower women in household decision-making, aligning with the Sustainable Development Goals (SDGs), particularly SDG 5 (Gender Equality) and SDG 10 (Reduced Inequalities). The evidence suggests that empowering younger women, especially those under 25, is essential for fostering decision-making capabilities that can enhance their roles in both domestic and professional spheres. Educational initiatives must play a central role, particularly in rural areas, by providing girls with access to quality education and culturally sensitive programs designed to promote autonomy and awareness of societal inequities [41]. Additionally, Economic empowerment by fostering education is a significant factor for independence in decision-making authority, especially for women. Skill development, job opportunities, and financial support for entrepreneurs are key to exercising decision-making power in their households. Addressing regional disparities through culturally sensitive interventions and collaboration with local organizations can promote self-reliance among women in rural and disadvantaged groups [42, 43]. Strengthening legal and policy frameworks to safeguard women’s rights in both household and public decision-making is indispensable. Policies must be regularly monitored and evaluated to ensure they address the evolving challenges of gender inequality effectively. By dismantling socio-economic and cultural barriers, the recommendations of this study aim to not only ensure greater autonomy for women but also contribute to a broader societal transformation. As Ban Ki-moon rightly noted, *“Gender equality and women’s empowerment are essential to achieving sustainable development”* [43]. Empowering women to take active roles in household decision-making is a step toward achieving gender parity and fostering equitable, inclusive progress in Indian households.
